# Revealing the Influence of SiC Particle Size on the Hot Workability of SiCp/6013 Aluminum Matrix Composites

**DOI:** 10.3390/ma16186292

**Published:** 2023-09-20

**Authors:** Shuang Chen, Changlong Wu, Guowei Bo, Haiyang Liu, Jie Tang, Dingfa Fu, Jie Teng, Fulin Jiang

**Affiliations:** 1 Hunan Provincial Key Laboratory of Vehicle Power and Transmission System, Hunan Institute of Engineering, Xiangtan 411104, China; chens20200528@126.com; 2 College of Materials Science and Engineering, Hunan University, Changsha 410082, China; wuchanglong@hnu.edu.cn (C.W.); tangj94@hnu.edu.cn (J.T.); hunu_fudingfa@163.com (D.F.); 3 College of Energy and Power Engineering, Changsha University of Science & Technology, Changsha 410114, China; guowei.bo@foxmail.com; 4 Hunan Province Engineering Research Center for the Preparation and Application of High Performance Aluminum Matrix Composites, Xiangxi 416100, China; 13974355549@163.com; 5 Hunan Everrich Composite Corp., Xiangxi 416100, China

**Keywords:** aluminum matrix composites, SiC particle size, hot workability, processing map, dynamic softening

## Abstract

SiC particle (SiCp) size has been found to significantly influence the hot workability of particle-reinforced aluminum matrix composites (AMC). In this work, therefore, three types of SiCp/6013 composites with different SiCp sizes (0.7, 5 and 15 μm) were prepared and then subjected to isothermal hot compression tests. In addition, constitutive analysis, processing maps and microstructural characterizations were used to reveal the influence of SiCp size on the hot workability of SiCp/6013 composite. The results showed that the values of hot deformation activation energy Q increased with decreasing SiCp size. Specifically, at lower temperatures (e.g., 350 and 400 °C), the highest peak stress was shown in the AMC with SiCp size of 0.7 μm (AMC-0.7), while in the AMC with SiCp size of 5 μm (AMC-5) at higher temperatures (e.g., 450 and 500 °C). This is because a finer SiCp size would lead to stronger dislocation pinning and grain refinement strengthening effects, and such effects would be weakened at higher temperatures. Further, dynamic softening mechanisms were found to transform from dynamic recovery to dynamic recrystallization with increasing SiCp size, and the dynamic recrystallization occurred more easily at higher temperatures and lower strain rates. Consequently, the instability zones of the composites are all mainly located in the deformation region with lower temperature and higher strain rate, and smaller SiCp results in larger instability zones.

## 1. Introduction

Particle-reinforced aluminum matrix composites (AMC) are widely used and indispensable in aerospace and automotive industries owing to their high specific strength, high specific stiffness, and high wear resistance [1,2,3]. Powder metallurgy is generally employed to prepare AMC because it is able to easily optimize the size and content of the reinforcing particles and increase the uniformity of distribution among reinforcing particles [4,5,6]. However, these AMCs prepared by powder metallurgy often require further hot working (e.g., hot rolling or extrusion) to reduce defects and increase the density [7,8,9]. Therefore, it is worthwhile to focus on studies that investigate the hot workability of AMC to better optimize processing parameters.

Due to the introduction of the hard reinforced particles, the hot deformation behavior of aluminum matrix composites has become more complex. In this regard, their hot workability, which indicates the ability of metallic materials to be deformed at elevated temperatures, could be evaluated using constitutive equations and processing maps. The constitutive equations generally describe the relationships between flow stress, strain rate and deformation temperature. Hence, the power or load required by hot forming processes as well as the flow mechanism of metals can be predicted through these equations. The processing map, which was first proposed by Prasad et al. based on the theory of dynamic material mode, can not only predict plastic deformation mechanisms under various conditions but can also help avoid unstable deformation [10,11,12,13,14,15]. For instance, it has been reported that the introduction of the hard reinforced particles is able to induce particle-stimulated nucleation and thus promote the occurrence of dynamic recrystallization in AMC [16,17,18,19,20]. In detail, by studying the microstructure evolution of hot extruded Al6061/SiCp composites, RAMESH et al. [20] found that the SiCp with size less than 5 μm can promote particle-excited recrystallization nucleation at low ln*Z* values. Likewise, Shao et al. [16] reported that during hot deformation, the dislocation generation rates in the 20 vol.% SiCp/2024Al composites prepared by powder metallurgy were higher around finer SiCp, and that the interfaces between 2024Al matrix and finer SiCp were the preferential sites for recrystallization nucleation. Further, by investigating the flow stress behavior of nano-SiCp/AZ91 composites during hot compression tests, Zhang et al. [17] revealed the effects of incorporated particles and their particle size on the hot workability of the studied alloy. The results showed that the incorporated SiC nanoparticles effectively increased the flow stress of the composites by blocking strain-induced dislocations, while the effect of micron SiC particles differed significantly due to competition between the pegging effect and particle-stimulated nucleation (PSN). Consequently, the added particles expand the instability regions of the studied composite to lower strain rates and higher temperatures. Similarly, the hot workability of Al-Si/SiCp + TiB_2_ hybrid aluminum matrix composites was also found to be strongly dependent on the contents of TiB_2_ in the work of Chen et al. [21]. The authors concluded that the area of the instability zones gradually increased with increasing TiB_2_ addition. Obviously, the size of the reinforced particle has an important impact on the mechanical properties and hot workability of composites [22,23,24,25]. However, most current studies mainly focus on the hot workability of the composite with specific content of particles, and studies on aluminum matrix composites with different sizes of single particles (especially for ceramic particles less than about 10 µm) are rarely reported. Therefore, the influence of the SiCp size with a wide range (from micron to submicron) on the hot workability of SiC particle-reinforced aluminum matrix composites should be paid more attention.

In this work, three types of SiC/6013 aluminum matrix composites with different particle sizes ranging from 0.7 to 15 µm were prepared, and the corresponding constitutive equations and hot processing maps were established based on hot compression tests. The effects of SiCp size on the microstructural evolution and deformation mechanism of the composites were revealed by microstructural observations.

## 2. Materials and Methods

**Figure 1:**
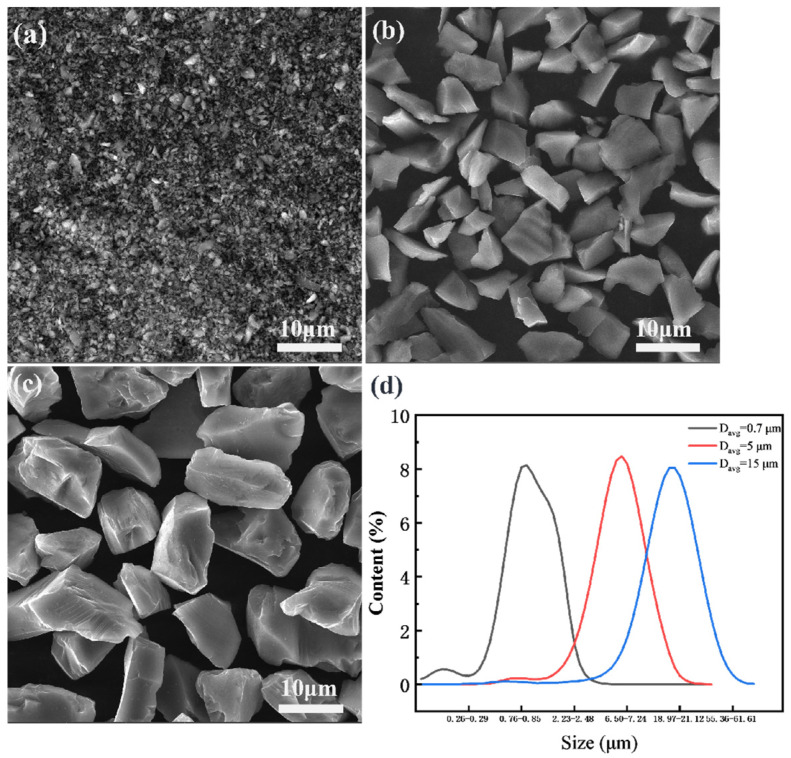
SEM images and particle size distribution of different SiC powders: (**a**) 0.7 μm; (**b**) 5 μm; (**c**) 15 μm; (**d**) Particle size distribution.

The commercial powder Al 6013 with an average size of 7 μm was selected as the matrix alloy, and SiCp powders with different particle sizes were used as the reinforced particles. Three types of SiCp/6013 aluminum matrix composites with their SiCp sizes of 0.7, 5 and 15 µm were prepared by powder metallurgy, and these three types of composites were denoted as AMC-0.7, AMC-7 and AMC-15, respectively. The particle sizes and ratios of these powders are shown in Table 1. The SEM images and particle size distribution of different SiC powders are shown in Figure 1. In detail, the weighed powders were placed into the ball mill tank and zirconium balls were added for ball milling and mixing. The mixing powder was cold isostatically pressed into a billet and then placed into a vacuum hot press machine for vacuum hot pressing and sintering at 580 °C and 75 MPa for 4 h. After that, the cylindrical ingots with a diameter of 85 mm and a length of 250 mm were obtained. To eliminate the defects, improve the interfacial bonding strength between the aluminum matrix and the reinforced particles, and refine the grain size, the composite ingots were then placed into the XJ-2500 hot extruder for extrusion at a temperature of 470 °C with an extrusion speed of 0.6 mm/s and an extrusion ratio of 13:1. Before extrusion, the ingots were held at 480 °C for 8 h. After extrusion, a 10 mm × 50 mm sheet was obtained. 

Each group of composites was then machined into cylindrical specimens with a diameter of 8 mm and a length of 10 mm for hot compression tests on a Gleeble-3500 thermal simulation machine. The isothermal continuous hot compression tests were conducted at 350 °C, 400 °C, 450 °C, and 500 °C with strain rates of 0.005 s^−1^, 0.05 s^−1^, 0.5 s^−1^, and 5 s^−1^ to a total strain of 0.7. To obtain a homogeneous temperature distribution before compression, all samples were heated to deformation temperature at a heating rate of 10 °C/s and held at that temperature for 3 min. Once compression ended, the specimens were immediately quenched in water [26].

For microstructural characterization, the undeformed samples were cut from the sheet along the extrusion direction, and the deformed samples were cut parallel to the compression direction along the centerline. Microstructural observations, i.e., scanning electron microscopy (SEM), electron backscatter diffraction (EBSD), and transmission electron microscopy (TEM), were implemented on a QUANTA200 SEM (FEI, Hillsboro, OR, USA) and a JEOL 2010 TEM (Tokyo, Japan) after preparing the specimens via conventional methods. In detail, the scanned samples were ground and mechanically and electrochemically polished, and parts of the samples were etched with a ratio of 20 vol.% perchloric acid and 80 vol.% anhydrous ethanol at a voltage of 20 V and a temperature of −25 °C to better observe the distribution of SiC particles. The TEM samples were first ground to 80 μm and then punched into disc with a diameter of 3 mm. The samples were then thinned using an anhydrous ethanol: nitric acid = 7:3 electrolyte with a Gatan 691 ion thinning instrument (Pleasanton, CA, USA).

## 3. Results and Discussions

### 3.1. Initial Microstructure

**Figure 2:**
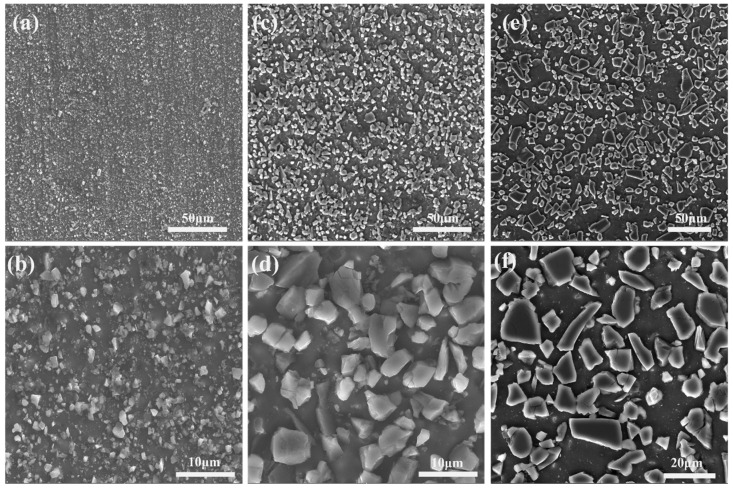
SEM secondary electron images showing the initial microstructures of SiCp/6013Al composites: (**a**,**b**) AMC-0.7; (**c**,**d**) AMC-5; (**e**,**f**) AMC-15.

**Figure 3:**
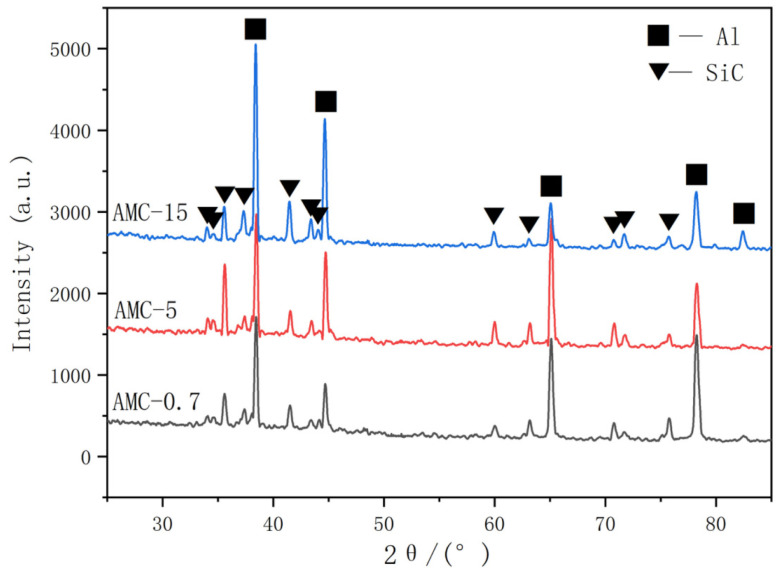
XRD patterns of three types of as-extruded SiCp/6013Al composites.

Figure 2 shows the initial microstructure of three types of SiCp/6013Al composites after extrusion. It can be seen that the SiCp in AMC-0.7 has a streamlined distribution along the extrusion direction, while the distribution of SiCp in AMC-5 and AMC-15 are more uniform and random. This is because the smaller SiC particles can flow and rotate more easily with the aluminum matrix during hot extrusion, whereas it is difficult for the larger SiC particles to flow and rotate with the aluminum matrix due to their higher resistance to deform. The larger magnification SEM images (Figure 2d–f) show that most of the SiC particles in the extruded SiCp/6013Al composites are intact without large-scale aggregation and fragmentation. The XRD patterns of three types of as-extruded SiCp/6013Al composite samples are shown in Figure 3. The peak values correspond to the Al phase and SiC phase, and no other phase peaks are clearly observed, which indicates that powder metallurgy and extrusion are suitable for the fabrication of SiCp/6013Al composites.

### 3.2. Flow Stress

**Figure 4:**
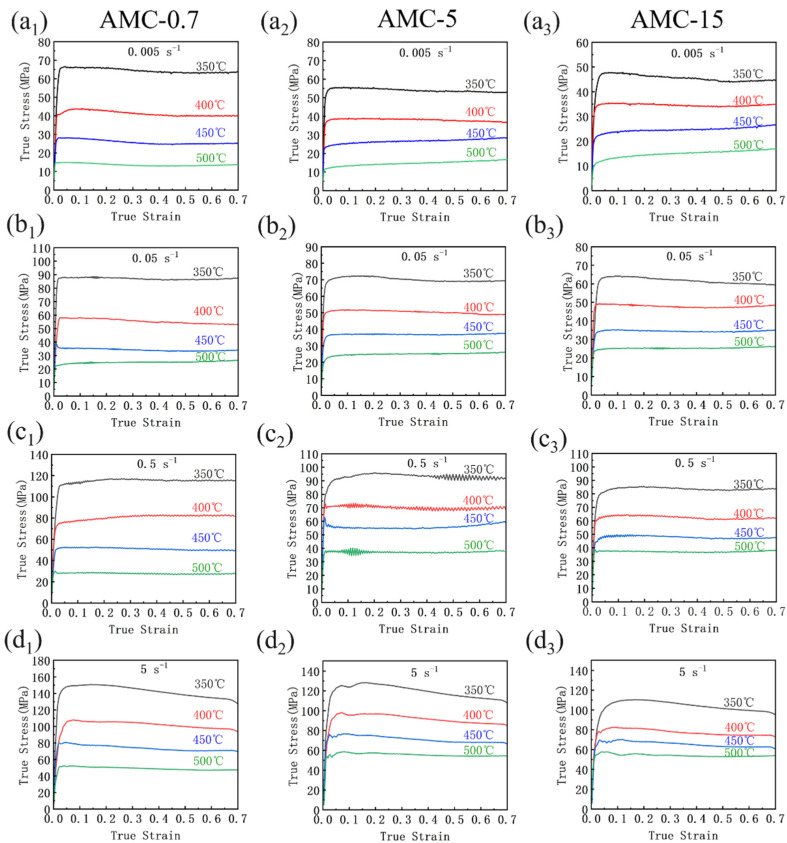
True stress–strain curves of AMC-0.7, AMC-5 and AMC-15 at different hot deformation conditions: (**a**) 0.005 s^−1^; (**b**) 0.05 s^−1^; (**c**) 0.5 s^−1^; (**d**) 5 s^−1^.

Figure 4 shows the true stress–strain curves of the three types of SiCp/6013Al composites. Clearly, with increasing strain, a rapid increase in flow stress to peak stress at the initial deformation stage followed by roughly unchanged trend are observed for all specimens. The steady-state stresses of the same composites decrease with increasing temperature and decreasing strain rate. However, the flow characteristics of the three composites were not identical at different deformation conditions. Specifically, it was found that the wave-like characteristics of the stress curves were more obvious for the three composites at a strain rate of 0.5 s^−1^, especially the AMC-5 composite. For instance, the flow stress of AMC-5 at the strain rate of 5 s^−1^ decreased and then increased after the peak stress. Such trends show the typical features of dynamic recrystallization. It has been suggested that after the initial dynamic recrystallization at the initial deformation stage, working hardening and dynamic recrystallization occur once again in a continuous cycle, leading to the wave-like stress–strain curve. The stress–strain curve with only one peak stress should only observed for the material which is only partially recrystallized. This indicates that the dynamic softening mechanism of the three composites varies at different deformation conditions and the size of SiC particles also have an obvious effect on the softening mechanism.

### 3.3. Peak Stress

**Figure 5:**
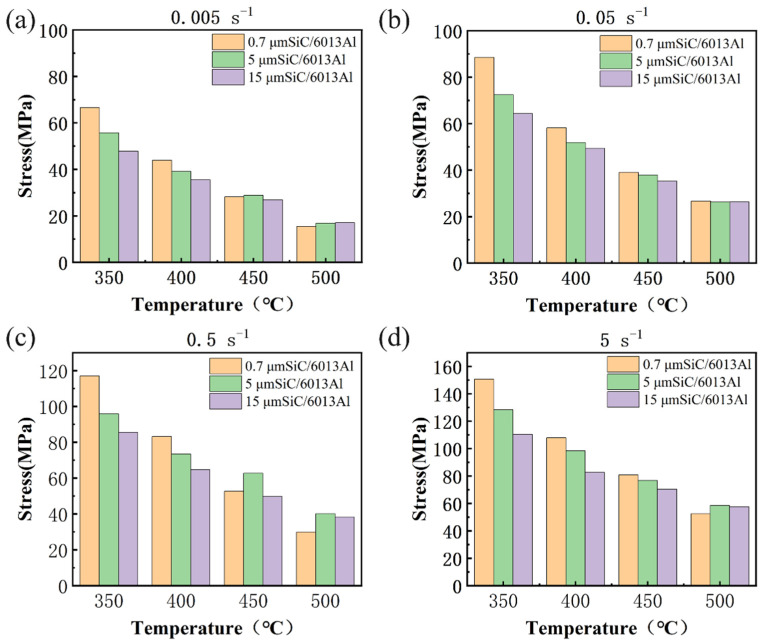
Peak stress of AMC-0.7, AMC-5 and AMC-15 under different hot deformation conditions: (**a**) 0.005 s^−1^; (**b**) 0.05 s^−1^; (**c**) 0.5 s^−1^; (**d**) 5 s^−1^.

Figure 5 summarizes the peak stresses of the three SiCp/6013Al composites. Obviously, when deformed at lower temperatures of 350 °C and 400 °C, the peak stress decreased with increasing SiCp size. This is because the finer SiC particles have a greater strengthening effect. However, when deformed at higher temperatures of 450 °C and 500 °C, the highest peak stresses were observed in different composites at different strain rate. For instance, the AMC-0.7 sample has the highest peak stress at 450 °C and 0.05 s^−1^, while the AMC-5 sample has the highest peak stress at 450 °C and 0.5 s^−1^. These phenomena should be related to their different softening mechanisms occurring. It is well known that the strengthening mechanism of SiC particle-reinforced aluminum matrix composites is mainly dislocation strengthening by blocking dislocation movement. Generally, the finer the SiCp size, the stronger the dislocation pinning strengthening. Moreover, the finer SiCp size causes grain refinement, thereby resulting in strengthening of grain boundaries. Therefore, different strengthening effects would be caused by different SiCp sizes and different interactions between dislocation and SiC particles at different temperatures. Specifically, at lower temperatures, the motion of dislocation is difficult and hence dynamic recovery is mainly responsible for the softening of the composites. In contrast, at high temperatures, the motivity of dislocation is increased the more grains are softened, leading to the occurrence of dynamic recrystallization. Because the degree of dynamic recrystallization was strongly dependent on strain rates at particular temperatures, the change trend of peak stress at higher temperatures shows irregularity.

### 3.4. Constitutive Equations

As mentioned before, the constitutive equations are able describe the relationships between flow stress, strain rate and deformation temperature. Hence, the power or load required by hot forming processes as well as the flow mechanism of metals can be predicted through these equations. Generally, the Arrhenius equation in hyperbolic sinusoidal form proposed by Sellars and Tegart [27] is widely used. The forms of the equations are as follows:

(1) $$\dot{\varepsilon }=A_{1}\sigma^{n_{1}}\exp\left(-\frac{Q}{RT}\right),\alpha \sigma <0.8$$

(2) $$\dot{\varepsilon }=A_{2}\exp\left(\beta \sigma \right)\exp\left(-\frac{Q}{RT}\right),\alpha \sigma >1.2\;$$

(3) $$\;\dot{\varepsilon }={A(\sinh\left(\alpha \sigma \right))}^{n}\exp\left(-\frac{Q}{RT}\right),for\;all\;\sigma \;$$

where $\dot{\varepsilon }$ is the strain rate in s^−1^; A_1_, A_2_, and A are structural factors; *σ* is the flow stress (MPa); *n*_1_ and *n* are stress indices; *α* is the stress level parameter; *Q* is the hot deformation activation energy (kJ/mol); *R* is the molar gas constant (8.31 J/mol K); *T* is the absolute temperature (K).

In general, the main equations for the low and high stress range can be described by Equations (1) and (2), respectively, and Equation (3) is applicable to the entire stress range. The effects of deformation temperature and deformation strain rate on stress can be coupled by the Zener–Hollomon (*Z*) parametric equation [28]. Substitution of Equation (3) into the *Z*-parameter equation yields the modified constitutive Equation (4) as follows:

(4) $$Z=\dot{\varepsilon }\exp\left(-\frac{Q}{RT}\right)={A(\sinh\left(\alpha \sigma \right))}^{n}$$

To find each parameter of the present constitutive equation, the following Equations (5)–(8) are obtained by taking the logarithm of both sides of Equations (1)–(4).

(5) $$\ln(\dot{\varepsilon })=\ln\left(A_{1}\right)+n_{1}\ln\sigma -\frac{Q}{RT}$$

(6) $$\ln(\dot{\varepsilon })=\ln\left(A_{2}\right)+\beta \sigma -\frac{Q}{RT}\;\;\;$$

(7) $$\ln(\dot{\varepsilon })=\ln A+n\ln\left[\sinh\left(\alpha \sigma \right)\right]-\frac{Q}{RT}$$

(8) $$\ln Z=\ln A+n\ln\left[\sinh\left(\alpha \sigma \right)\right]\;$$

The derivative of Equation (7) leads to the formula for calculating the value of *Q*:

(9) $$Q=R{\left.\frac{\partial \ln\left[\sinh\left(\alpha \sigma \right)\right]}{\partial \left(1/T\right)}\right|}_{\dot{\varepsilon }}{\left.\frac{\partial \dot{\ln\varepsilon }}{\partial \ln\left[\sinh\left(\alpha \sigma \right)\right]}\right|}_{T}=Rnk\;$$

The calculation steps for each parameter are as follows:

(1) Calculate the value of *n*_1_ and plot the coordinates of ln *σ*–ln $\dot{\varepsilon }$. ln*σ* is linearly related to ln $\dot{\varepsilon }$, and use the linear equation to fit the *n*_1_ as the average slope of the ln *σ*–ln $\dot{\varepsilon }$ plotting.

(2) Calculate the value of *β* and plot the coordinates of *σ*–ln $\dot{\varepsilon }$. *σ* is linearly related to ln $\dot{\varepsilon }$ and the linear equation is fitted to yield *β* as the mean value of the slope in the *σ*–ln $\dot{\varepsilon }$ plot.

(3) Calculate the value of *α*_1_ according to the formula *α*_1_ = *n*/*β*.

(4) Calculate the value of *Q* and plot the coordinates of ln [sinh(*α*_1_*σ*)]–ln $\dot{\varepsilon }$, which allows to find the average value of the slope *n*_2_, and then calculate the value of the adjusted *α*_2_ according to the formula *α* = *n*/*β* and plot the ln[sinh(*α*_2_*σ*)]–ln $\dot{\varepsilon }$ coordinate again to obtain the value of *n*_3_. After that, plot the coordinate plot of 1000/*T*–ln[sinh(*α*_2_*σ*)] to obtain the value of *k*_1_. We then have *Q*_1_ = R*n*_3_*k*_1_, and to obtain more accurate values, the iterations can be repeated to obtain *Q*_2_ = R*n*_4_*k*_2_ and *Q*_3_ = R*n*_5_*k*_3_.

The parameters of the constitutive equations of the three composites calculated by the above steps are shown in Table 2. Clearly, it can be seen that the SiCp size has a significant influence on the *Q* value of the composite. In detail, the hot deformation activation energy (*Q*) values for AMC-0.7, AMC-5 and AMC-15 are estimated to be 237.08, 170.61 and 161.07 kJ/mol, respectively. The values of *Q*, which commonly serves as an indicator of the degree of deformation difficulty during hot working, coincide with the sequence of flow stresses in Figure 3. Moreover, the difference in the *Q* value between AMC-0.7 and AMC-5 is greater than that between AMC-5 and AMC-15. This can be explained by the Orowan strengthening mechanism. Specifically, the finer the size of the second phase and the smaller the particle spacing, the stronger the strengthening effect [29,30]. Therefore, the particle strengthening effect is more pronounced from micron to submicron size of the reinforced particles. In other words, the number of particles increases more for the same mass of SiC particles from 0.7 μm to 5 μm than for that from 15 μm to 5 μm.

The constitutive equations of the AMC-0.7, AMC-5 and AMC-15 can be obtained as (10), (11) and (12), respectively:

(10) $$\dot{\varepsilon }={1.095\times 10^{12}[\sinh\left(0.034788729\sigma \right)]}^{3.2185925}\exp\left(-\frac{237080}{RT}\right)$$

(11) $$\dot{\varepsilon }={1.607\times 10^{10}[\sinh\left(0.034200002\sigma \right)]}^{3.3426775}\exp\left(-\frac{170607}{RT}\right)\;\;\;$$

(12) $$\dot{\varepsilon }={3.085\times 10^{9}[\sinh\left(0.035860451\sigma \right)]}^{3.6877}\exp\left(-\frac{161069}{RT}\right)\;$$

The above equation can also be converted into an equation with *Z*-value (13):

(13) $$\sigma =\frac{1}{\alpha }\ln\left\{{\left(\frac{Z}{A}\right)}^{\frac{1}{n}}+{\left[{\frac{Z}{A}}^{\frac{2}{n}}+1\right]}^{\frac{1}{2}}\right\}$$

**Figure 6:**
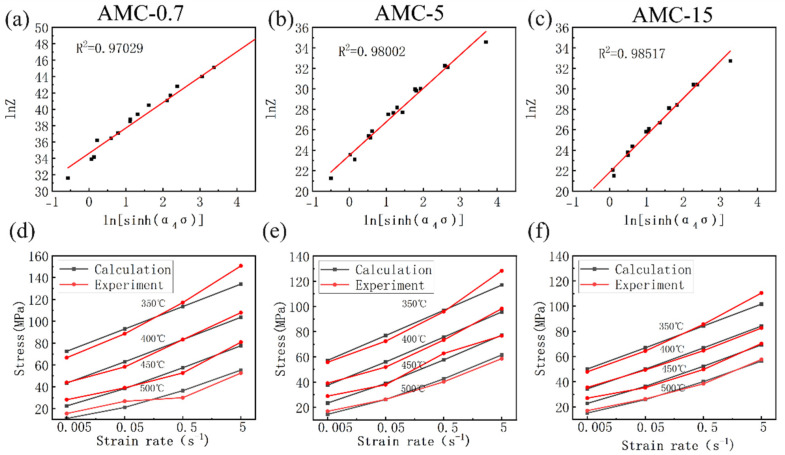
(**a**–**c**) The relationship between ln*Z* and ln[sinh(*α*_4_*σ*)] for AMC-0.7, AMC-5 and AMC-15; (**d**–**f**) Calculated and experimental values of peak stresses for AMC-0.7, AMC-5 and AMC-15.

Figure 6a–c show the relationship between ln*Z* and ln[sinh(*α*_4_*σ*)] for AMC-0.7, AMC-5 and AMC-15 with linearly fitted correlation coefficients of 0.97029, 0.98002, and 0.98517, respectively. These high correlation coefficients indicate the reliability of the Zener–Hollomon parametric equation in hyperbolic sinusoidal form. In addition, the comparisons between the experimental values of peak stresses and the calculated values of the intrinsic model are shown in Figure 6d,e. It can be seen that the modeling results show good agreement with the experimental values of the three composites. Therefore, the obtained constitutive equations in hyperbolic sinusoidal form could be used to well predict flow stresses of the three composites.

### 3.5. Processing Maps

The hot processing map, which is based on the dynamic material model (DMM), generally consists of a power dissipation diagram and a rheological instability diagram superimposed [31,32]. According to the dynamic material model theory, the external output energy *P* consists of the dissipative quantity (*G*) and the dissipative co-efficient (*J*), as shown below [33]:

(14) $$P=\sigma \dot{\varepsilon }=G+J=\int_{0}^{\dot{\varepsilon }}\sigma d\dot{\varepsilon }+\int_{0}^{\sigma }\dot{\varepsilon }d\sigma \;\;$$

where *G* represents the energy consumed by plastic deformation, and is mostly in the form of hot energy with a small amount of energy stored inside the crystal in the form of crystal defect energy. *J* represents the energy required for the material to undergo microstructural evolution. When the strain and deformation temperature are constant, the ratio of *J* and *G* can be defined in terms of the strain rate sensitivity index *m*:

(15) $$m=\frac{\partial J}{\partial G}=\frac{\dot{\varepsilon }\partial \sigma }{\sigma \partial \dot{\varepsilon }}={\left.\frac{\partial (\ln\sigma )}{\partial (\ln\dot{\varepsilon })}\right|}_{T,\varepsilon }\;\;$$

Defining *J*/*J*_max_ as the power dissipation factor *η*, the formula for *η* is:

(16) $$\eta =\frac{J}{J_{max}}=\frac{2m}{1+m}\;\;$$

The physical implication of *η* is the ratio of energy consumed for microstructural transformation during deformation to the ideal linear dissipation energy. Larger power dissipation factor *η* represents higher efficient energy used for microstructural changes during deformation, further indicating better hot workability. Based on the nonlinear correlation between the value of the strain rate sensitivity index m and the strain rate $\dot{\varepsilon }$, the instability parameter *ξ* in the instability diagram can be defined as follows [33,34]:

(17) $$\xi (\dot{\varepsilon })=\frac{\partial \ln\left[m/(m+1)\right]}{\partial \ln\dot{\varepsilon }}+m<0\;$$

By calculating the value of *ξ* at different temperatures and different strain rates, a rheological instability diagram can be drawn. The physical implication of the instability criterion is that the entropy value generated by the system is lower than the strain rate, which will cause local rheology of the system in terms of defects such as holes, cracks or adiabatic shear zones. Therefore, the experimental temperature and strain rate located in the domains with negative values of *ξ* are ‘’unsafe’’ for hot working due to flow instability. 

**Figure 7:**
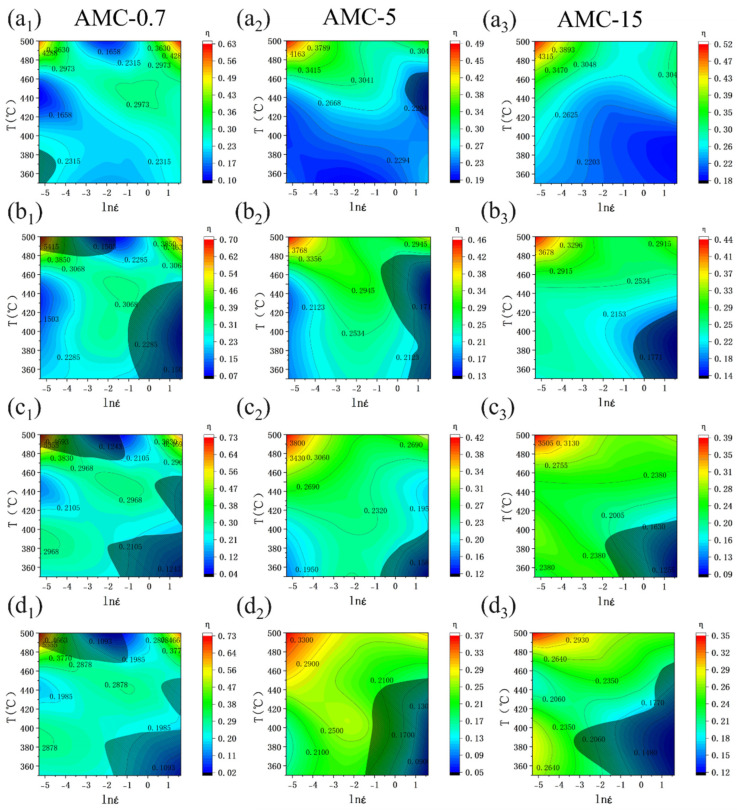
Processing maps of AMC-0.7, AMC-5 and AMC-15 at different strain levels: (**a**) ε = 0.1, (**b**) ε = 0.3, (**c**) ε = 0.5, (**d**) ε = 0.7.

Based on the above principles, the processing map for the three composites can be established by superimposing the instability map over the power dissipation map, as shown in Figure 7. The instability region is marked with shaded parts in the instability map, while the power dissipation map is displayed using colored contour maps. Different colors represent different dissipation efficiencies. For instance, the dissipation efficiency decreases from red to blue contour. The distribution of the instability zone and its change with strain for the three composites display the same trend as the evolution of peak stress in Figure 6. In detail, the instability zones are mainly located in the low-temperature and high-strain rate region, and their areas all show an increasing trend with increasing strain. By comparing the instability zone of the studied three composites with different SiCp sizes, it can be found that the finer the SiC, the larger the area of instability zone at the same strain. This indicates that the increasing SiCp size would make it easier for the composite to deform and for local instability to occur less readily. This may be due to the fact that when the composite is hot deformed, the strain is mainly accumulated in the aluminum matrix with higher ductility. Consequently, the larger the size of SiC particles with the same mass fraction means that the number of SiC particles is lower and the resistance to the flow of the aluminum matrix is also weaker. Meanwhile, the finer the SiC particles, the greater the interface area between them and the aluminum matrix, thus increasing the possibility of defects at the interface. As a result, the aluminum matrix flows with more ease. The value distributions of the power dissipation efficiency *η* show that the high *η* values for all three composites are mainly located in the region of higher temperature and lower strain rate. Therefore, the preferred hot processing parameters for SiCp/6013Al composites are (460–490 °C, 0.005–0.5 s^−1^), and the strain during single-pass deformation should not exceed 0.3 because such processing interval is less prone to defects and has higher processing efficiency. 

### 3.6. Microstructural Characterization

#### 3.6.1. Observation of Hot Deformation Instability Characteristics

**Figure 8:**
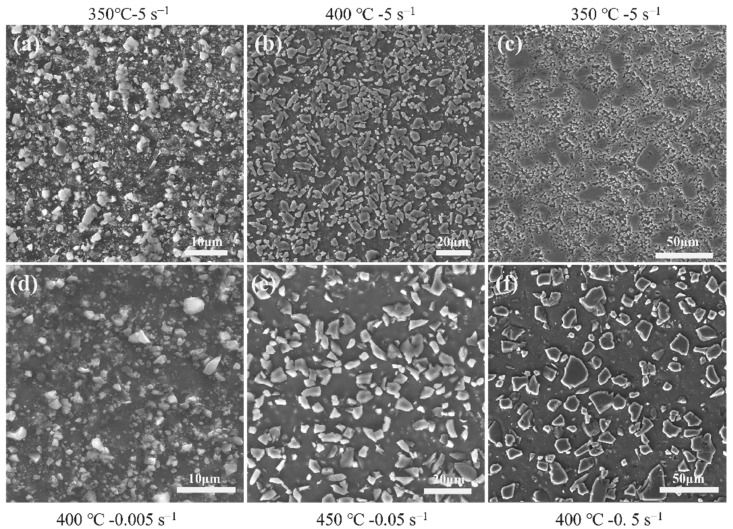
SEM images of the three composites deformed at instability and safety zones with a strain of 0.7: instability zones: (**a**) AMC-0.7; (**b**) AMC-5; (**c**) AMC-15; safety zones: (**d**) AMC-0.7; (**e**) AMC-5; (**f**) AMC-15.

To verify the reliability of the processing map, SEM observations were performed on the three composites deformed under different conditions, as shown in Figure 8. Obviously, in Figure 8a–c which show the instability microstructure of the composites deformed in instability zones, the microstructure is characterized by the aggregation of fragments due to the fragmentation of SiC particles. Such defects would remarkably affect the mechanical properties of the composite, decreasing the flow stress of the composites. Moreover, the fragmentation of SiC particles becomes more severe with increasing SiCp size. Figure 8d–f show the microstructures of samples deformed within corresponding safety zones. It can be seen that the SiC particles in the safety zones remain relatively intact, and the interface between the particles and the aluminum matrix is clear. In addition, there is no obvious aggregation phenomenon. This is because the higher temperature provides more thermal activation energy and softens the aluminum matrix and lower strain rate could provide the SiC particles enough time to rotate with the aluminum matrix to coordinate the hot deformation of the composites.

#### 3.6.2. Dynamic Softening Mechanism

**Figure 9:**
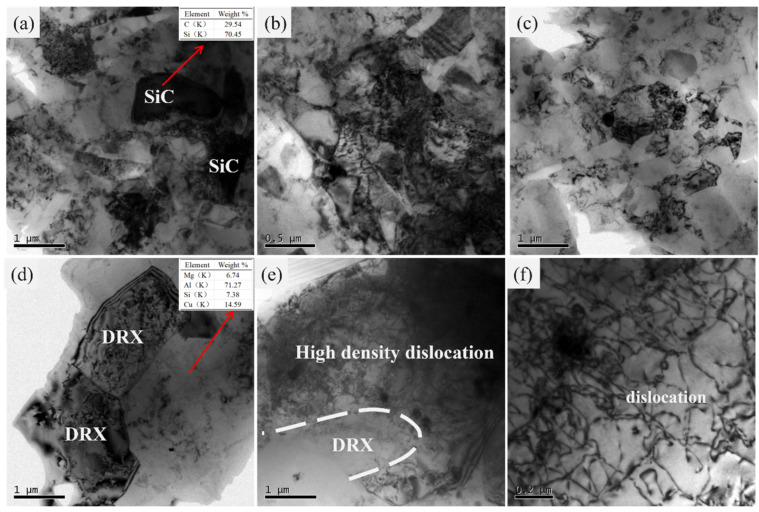
TEM images of AMC-0.7 samples deformed at different deformation conditions (ln*Z* values): (**a**–**c**) 350 °C-0.5 s^−1^ (ln*Z* = 45.10); (**d**–**f**) 500 °C-0.05 s^−1^ (ln*Z* = 33.91).

To investigate the effect of SiCp size on the deformation mechanism of SiCp/6013Al composites during hot compression, some AMC-0.7 and AMC-15 samples were selected for TEM observation. Figure 9 shows the TEM images of the AMC-0.7 deformed with different deformation conditions (namely different ln*Z* values). In Figure 9a–c, a large number of fine subgrains were observed in the AMC-0.7 samples deformed at 350 °C and 0.5s^−1^ (ln*Z* = 45.10). The subgrain size is generally less than 1 μm and some subgrains are accompanied by high-density dislocations. Similar dislocation tangles are also observed around the SiC particles. These abundant subgrains indicate that the main dynamic softening mechanism of AMC-0.7 at a lower temperature and higher strain rate is dynamic recovery (DRV). In principle, dynamic recovery describes the integrated functions of the accumulation and interactions of dislocations, migration of dislocations or subgrain boundaries. Generally, with increasing strain level, many dislocations gradually tangle to form a cellular wall. These cellular structures then further develop to become subgrain and/or grain boundaries. With further climbing and sliding of dislocations, subgrains polymerize into grains. As a result, the dislocation density decreases remarkably, leading to a drop in flow stress. Therefore, the working hardening rate and dynamic softening rate would reach dynamic equilibrium at a critical strain, leading to the steady-state flow stress. When deformed at low ln*Z* (500 °C and 0.05 s^−1^, ln*Z* = 33.91), as shown in Figure 9d–f, two neighboring recrystallized grains with clear boundary were clearly observed in the AMC-0.7 specimen (Figure 9d). In Figure 9e, the ambiguous grain boundary can be observed, showing the nucleation of a recrystallized grain which has not formed clearly at this time. Such results can also be confirmed by the high density of dislocation cells in Figure 9e. In addition, it can also be seen that the black particles which should be *α*(AlFeMnSi) phase have a strong pinning effect on dislocation movement, resulting in higher dislocation density around the phase. Therefore, it can be concluded that the dynamic recovery and recrystallization are the main dynamic softening mechanisms of AMC-0.7 at higher ln*Z* values (higher strain rate and lower temperature) and lower ln*Z* values (lower strain rate and higher temperature), respectively.

**Figure 10:**
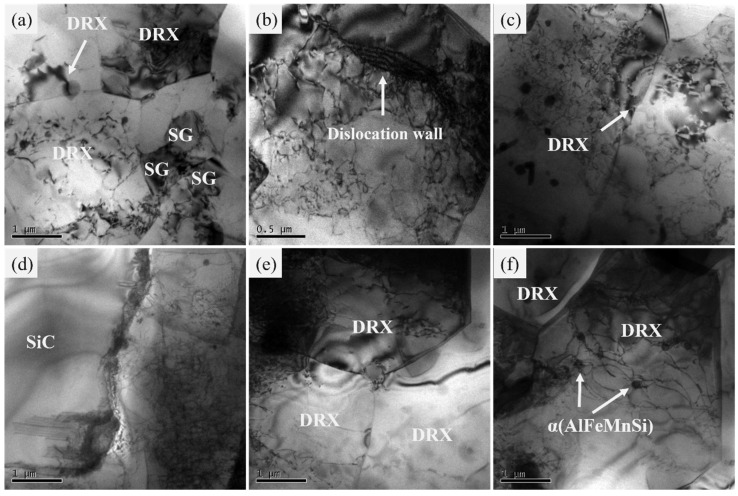
TEM images of the AMC-15 samples deformed under different deformation conditions (ln*Z* values): (**a**–**c**) 350 °C-0.5 s^−1^ (ln*Z* = 32.72); (**d**–**f**) 500 °C-0.05 s^−1^ (ln*Z* = 22.08).

The TEM images of AMC-15 samples deformed under different deformation conditions (ln*Z* values) are shown in Figure 10. When deformed at 350 °C and 0.5 s^−1^ (ln*Z* = 32.72, as shown in Figure 10a–c), a few subgrains (SG) and recrystallized grains as well as the nucleation of recrystallized grains at the grain boundaries are observed in Figure 10a, indicating the incomplete occurrence of dynamic recrystallization. Likewise, in Figure 10b, dislocation walls which can be considered as the grain boundary between the recrystallized grains are obviously detected. Generally, dynamic recrystallization can also be divided into discontinuous dynamic recrystallization (Discontinuous DRX, DDRX), continuous dynamic recrystallization (Continuous DRX, CDRX), and geometric dynamic recrystallization (Geometric DRX, GDRX). In detail, the process of nucleation of new strain-free grains that grow in dislocation-filled regions is frequently considered as the typical feature of discontinuous dynamic recrystallization. Continuous dynamic recrystallization refers to a process where clear dislocation cells or substructures with low-angle grain boundaries (LAGBs) evolve into high-angle grain boundaries (HAGBs) during large deformations. Geometric dynamic recrystallization, on the other hand, indicates that the elongated grains are cut off and new fine grains are formed in large strain stage [35]. In this work, the recrystallization characteristics in Figure 10a–c show that continuous dynamic recrystallization occurred in the AMC-15 samples. Specifically, the cross-slip of dislocations in the non-basal plane is activated near the original grain boundary where the stress is highly concentrated, thereby changing the dislocation slip direction into edge orientation. Meanwhile, due to the relatively high fault energy of the aluminum matrix, this cross-slip makes it easier for the edge-type dislocations to climb. Further, the cross-slip and the climbing dislocations rearrangement lead to the formation of dislocation network with low-angle grain boundaries near the original boundary. After that, the continuous absorption of dislocations within the low-angle grain boundary leads to the occurrence of CDRX [35,36]. Figure 10d–f showed the TEM micrographs of AMC-15 specimen deformed at 500 °C–0.05 s^−1^ (ln*Z* = 22.08). In Figure 10d, the morphology of the interface between the large-size SiC particles and the aluminum matrix showed that the large-size SiC particles are not tightly bonded to the aluminum matrix, and there is also partial dislocation tangling near the SiC particles. In Figure 10e, the recrystallized grains have grown larger in size. Meanwhile, clear interactions between dislocation/substructures and *α* (AlFeMnSi) relative dislocations can be observed in Figure 10f. Therefore, it can be concluded that the dynamic recovery with partial dynamic recrystallization are the main dynamic softening mechanisms of AMC-15 at higher ln*Z* values (higher strain rate and lower temperature), while a higher degree of dynamic recrystallization is mainly responsible for the dynamic softening at lower ln*Z* values (lower strain rate and higher temperature).

According to the classical Particle Simulated Nucleation (PSN) mechanism [19,20,37], the presence of SiC particles in the composites could increase the stored strain dislocation energy of the aluminum matrix after deformation, further leading to the occurrence of dynamic recrystallization during plastic deformation. When the particle size is larger than 1 μm, PSN can easily occur because SiC particles would not take part in plastic deformation of the composites. Hence, the deformation zone near the particles with high dislocation density and large lattice orientation difference would be formed in the matrix, thus changing the deformed subgrains into the recrystallization core and accelerating DRX [37]. In contrast, although dynamic recrystallization can also occur in the sample with finer SiCp size (AMC-0.7), the pinning effect of finer SiC particles on dislocation and subgrain boundary movement would result in the occurrence of DRX of the AMC-0.7 specimen only under higher temperatures and lower deformation rates. This is because higher temperatures facilitate the movement of dislocations and subgrain boundaries, and a low strain rate could provide enough time for subgrains to migrate and grow. Moreover, the larger the SiCp size, the larger the particle spacing and the lower the resistance to dislocation and grain boundary movement. In other words, the migration of subgrains and the growth of DRX grains would be easier. Accordingly, larger DRX grains can be observed at lower strain rates and higher temperatures. Further, by comparing the ln*Z* values of the four samples in Figure 8 and Figure 9, it was found that DRV occurred in the sample with ln*Z* of 45.10, while DRX was mainly in the samples with ln*Z* of 33.91, 32.72, and 22.08, as shown in Table 3. And according to Figure 8 and Figure 9, the smaller the ln*Z* value, the higher the degree of DRX, which indicates that the dynamic softening mechanism of the studied composites could be roughly judged by the size of the ln*Z* value. In detail, the larger the ln*Z* value, the lower the number of recrystallized grains and the higher the number of substructures in the composites, showing higher degree of DRV and higher flow stress.

## 4. Conclusions

In this study, three types of SiCp/6013 composites with different SiCp sizes (0.7, 5 and 15 μm) were prepared and then subjected to isothermal hot compression tests. Subsequently, the influence of SiCp size on the hot deformation behavior of SiCp/6013Al composites was investigated by means of constitutive analysis, processing map and microstructural characterization. The main conclusions were obtained as follows:

1. Smaller SiCp in composites results in higher peak stresses at lower deformation temperatures. However, when deformed at higher temperatures, the highest peak stress was shown in the AMC with SiCp size of 5 μm (AMC-0.7 composite) due to the softening of grain boundaries and weakening of the grain refinement strengthening caused by SiCp at high temperatures.
2. The flow stresses of the AMC-0.7, AMC-5 and AMC-15 could be well described by a Zener–Hollomon parameter in the hyperbolic-sine equation with the hot deformation activation energies *Q* of 237.08, 170.61 and 161.07 kJ/mol, respectively. And the value of Q, which represents the plastic deformation resistance, increased with decreasing SiCp size.
3. The instability zones of the composites are all mainly located in deformation region with lower temperature and higher strain rate. The area of the instability zone increased with increasing strain and decreasing SiCp size. The optimal hot processing parameters for SiCp/6013Al composites are (460–490 °C, 0.005–0.5 s^−1^), and the total strain of a single-pass deformation should not exceed 0.3.
4. The dynamic softening mechanisms of the AMC-0.7 were DRV at lower temperature and higher strain rate, while coupled effect partial DRV and partial DRX at higher temperature and lower strain rate. Likewise, DRV with partial DRX were responsible for the dynamic softening of AMC-15 at lower temperature and higher strain rate and at higher temperature and low strain rate, respectively. Therefore, it was concluded that the softening mechanism of the composites would transform from DRV to DRX with increasing SiCp size, and the larger the SiCp size, the easier the nucleation and growth of DRX.

## Figures and Tables

**Table 1:** Powder sizes and proportions of the composites.

Composites	6013Al Powder Particle Size (μm)	6013Al Content (wt.%)	SiCp Size (μm)	SiCp Content (wt.%)
AMC-0.7	7	80	0.7	20
AMC-5	7	80	5	20
AMC-15	7	80	15	20

**Table 2:** Parameters of the constitutive equations of the composites.

Composites	*n*	*α* (MPa^−1^)	*Q* (kJ/mol)	A (s^−1^)
AMC-0.7	*n*_1_ = 7.083715			1.095 × 10^12^
	*n*_2_ = 4.969192	*α*_1_ = 0.01748023		
	*n*_3_ = 4.073672	*α*_2_ = 0.02491853	*Q*_1_ = 232.855023	
	*n*_4_ = 3.559342	*α*_3_ = 0.03039640	*Q*_2_ = 235.254364	
	*n*_5_ = 3.218592	*α*_4_ = 0.03478872	*Q*_3_ = 237.079871	
AMC-5	*n*_1_ = 6.92382			1.607 × 10^10^
	*n*_2_ = 4.98347	*α*_1_ = 0.01811355		
	*n*_3_ = 4.151257	*α*_2_ = 0.02516619	*Q*_1_ = 169.570791	
	*n*_4_ = 3.667105	*α*_3_ = 0.03021132	*Q*_2_ = 170.134270	
	*n*_5_ = 3.342677	*α*_4_ = 0.03420000	*Q*_3_ = 170.606507	
AMC-15	*n*_1_ = 7.306857			3.085 × 10^9^
	*n*_2_ = 5.355895	*α*_1_ = 0.019732491		
	*n*_3_ = 4.514075	*α*_2_ = 0.026920337	*Q*_1_ = 160.7750554	
	*n*_4_ = 4.020655	*α*_3_ = 0.031940652	*Q*_2_ = 160.9388728	
	*n*_5_ = 3.6877	*α*_4_ = 0.035860451	*Q*_3_ = 161.0694601	

**Table 3:** The ln*Z* values of different samples.

Composites	Deformation Parameters	ln*Z*
AMC-0.7	350 °C–0.5 s^−1^	45.10
	500 °C–0.05 s^−1^	33.91
AMC-15	350 °C–0.5 s^−1^	32.72
	500 °C–0.05 s^−1^	22.08

## Data Availability

Not applicable.
